# Mitigation of salt stress in *Sorghum bicolor* L. by the halotolerant endophyte *Pseudomonas stutzeri* ISE12

**DOI:** 10.3389/fpls.2024.1458540

**Published:** 2024-09-23

**Authors:** Ahmad Rajabi Dehnavi, Agnieszka Piernik, Agnieszka Ludwiczak, Sonia Szymańska, Anna Ciarkowska, Stefany Cárdenas Pérez, Katarzyna Hrynkiewicz

**Affiliations:** ^1^ Department of Geobotany and Landscape Planning, Faculty of Biological and Veterinary Sciences, Nicolaus Copernicus University in Toruń, Toruń, Poland; ^2^ Department of Microbiology, Faculty of Biological and Veterinary Sciences, Nicolaus Copernicus University in Toruń, Toruń, Poland; ^3^ Department of Biochemistry, Faculty of Biological and Veterinary Sciences, Nicolaus Copernicus University in Toruń, Toruń, Poland

**Keywords:** endophytic bacteria, halophyte, halotolerant bacteria, plant growth promoting bacteria, salt stress, *Sorghum bicolor* L. Moench

## Abstract

Increasing soil salinity, exacerbated by climate change, threatens seed germination and crop growth, causing significant agricultural losses. Using bioinoculants based on halotolerant plant growth-promoting endophytes (PGPEs) in modern agriculture is the most promising and sustainable method for supporting plant growth under salt-stress conditions. Our study evaluated the efficacy of *Pseudomonas stutzeri* ISE12, an endophyte derived from the extreme halophyte *Salicornia europaea*, in enhancing the salinity tolerance of sorghum (*Sorghum bicolor* L.). We hypothesized that *P. stutzeri* ISE12 would improve sorghum salt tolerance to salinity, with the extent of the increase in tolerance depending on the genotype’s sensitivity to salt stress. Experiments were conducted for two sorghum genotypes differing in salinity tolerance (Pegah - salt tolerant, and Payam - salt sensitive), which were inoculated with a selected bacterium at different salinity concentrations (0, 100, 150, and 200 mM NaCl). For germination, we measured germination percentage and index, mean germination time, vigor, shoot and root length of seedlings, and fresh and dry weight. In pot experiments, we assessed the number of leaves, leaf area, specific leaf area, leaf weight ratio, relative root weight, plantlet shoot and root length, fresh and dry weight, proline and hydrogen peroxide concentrations, and peroxidase enzyme activity. Our study demonstrated that inoculation significantly enhanced germination and growth for both sorghum genotypes. The salinity-sensitive genotype (Payam) responded better to bacterial inoculation during germination and early seedling growth stages, showing approximately 1.4 to 1.8 times greater improvement than the salinity-tolerant genotype (Pegah). Payam also displayed better performance at the plantlet growth stage, between 1.1 and 2.6 times higher than Pegah. Furthermore, inoculation significantly reduced hydrogen peroxide, peroxidase activity, and proline levels in both sorghum genotypes. These reductions were notably more pronounced in Payam, with up to 1.5, 1.3, and 1.5 times greater reductions than in Pegah. These results highlight the efficacy of *P. stutzeri* ISE12 in alleviating oxidative stress and reducing energy expenditure on defense mechanisms in sorghum, particularly benefiting salt-sensitive genotypes. Our findings highlight the potential of the bacterial endophyte *P. stutzeri* ISE12 as a valuable bioinoculant to promote sorghum growth under saline conditions.

## 1 Introduction

Salinity is one of the most important abiotic stresses that affect the plants’ growth, development, and final yield, especially in arid and semi-arid areas (Alotaibi et al., 2023). It has been reported that about 20% of irrigated and 2% of dry land areas are affected by salinity (Lekka et al., 2023). Plants’ response to salinity is complex and related to changes in all physiological, morphological, biochemical, and metabolic pathways (Mashabela et al., 2023). Salt stress causes inhibition of crop growth and development mainly by osmotic stress and ionic toxicity (Ludwiczak et al., 2021; Fu and Yang, 2023). These effects lead to nutrient imbalance, enzymatic and metabolic inhibition, and alteration levels of growth regulators (Colin et al., 2022). Furthermore, under saline conditions, the generation of Reactive Oxygen Species (ROS), such as superoxide radicals, hydrogen peroxide, hydroxyl radicals, and singlet oxygen in chloroplasts, mitochondria, and apoplectic space, causes oxidative stress and damage to membranes, nucleic acids, cell membranes, cellular structure, and ion leakage (Pirasteh-Anosheh et al., 2023). These adverse effects cause a reduction in the quality and yield of crops (Truşcă et al., 2023). Nevertheless, these negative effects depend on the severity and duration of stress, the genetic properties of plants, and other environmental factors. Plant species deploy a range of protective mechanisms to mitigate salt stress. These include the scavenging of ROS through antioxidant pathways, prominently involving enzymes like peroxidases (Hasanuzzaman et al., 2021). Furthermore, they employ osmoregulatory compounds such as proline to stabilize and protect macromolecules and cellular organelles (Pirasteh-Anosheh et al., 2023).

The global problem of increasing saline areas and the growing demand for food force us to look for new and effective methods of reclaiming areas for food crops. One of the sustainable crop strategies being considered is using endophytes isolated from halophytic plants. Numerous halotolerant/halophytic plant growth-promoting endophytic bacteria could directly interact with the host plant. Many known endophytic *Pseudomonas* spp. strains exhibit significant positive effects on stress alleviation and growth promotion across various crops, supported by extensive research (Costa-Gutierrez et al., 2022; Szymańska et al., 2022). These bacteria establish beneficial associations with plants by colonizing their rhizosphere, employing diverse mechanisms to enhance growth and stress tolerance (Uyi et al., 2024). Certain *Pseudomonas* species have been observed to solubilize phosphate, fix nitrogen, and produce phytohormones (Teotia and Chaudhary, 2024). These activities collectively stimulate root growth and enhance nutrient uptake in plants. They can also synthesize siderophores, compete against pathogens, and induce systemic resistance in plants (Saleem et al., 2024). It is noteworthy that *P. stutzeri* stands out for its abilities as a biofertilizer, especially under saline conditions, increasing growth and salinity tolerance in different plant species (Lami et al., 2020; Sah et al., 2021; Szymańska et al., 2022; Zheng et al., 2024). In our previous studies, we noted positive effects of inoculation with the endophytic strain *P. stutzeri* ISE12 isolated from the extreme halophyte *Salicornia europaea* on the growth of *Beta vulgaris* (fodder and red beet) (Piernik et al., 2017; Szymańska et al., 2020), *Brassica napus* (rapeseed) (Szymańska et al., 2019), *Hordeum vulgare* (barley), *Lactuca sativa* (lettuce) and *Helianthus annuus* (common sunflower) (Szymańska et al., 2022). These findings collectively underscore the potential of *P. stutzeri* ISE12 as an effective bioinoculant for enhancing plant performance and resilience in saline environments.

Sorghum (*Sorghum bicolor* L.) is an annual C4 crop and one of the five most important crops around the world that is used as human food and livestock forage in more than 98 countries around the world (Iqbal, 2015; Khoddami et al., 2023). This species is more economical than other cereal forages due to fewer supplies of irrigation and fertilizers. This crop is mainly cultivated in arid and semi-arid areas with high temperatures and low rainfall (Iqbal, 2015; Khoddami et al., 2023). In addition, sorghum can be used in areas with high salinity (Khoddami et al., 2023; Liaqat et al., 2024). However, it has been observed that the response of different sorghum genotypes to salinity differs (Mansour et al., 2021; Rajabi Dehnavi et al., 2023). The varying tolerance of different sorghum genotypes to salinity can be attributed to genetic diversity, which influences their ability to regulate ion uptake and transport, maintain cellular osmotic balance, and activate stress-responsive mechanisms. Several studies have explored the role of *Pseudomonas* spp. in enhancing sorghum growth under saline conditions. For instance, research demonstrated that *Pseudomonas mohnii* and *Pseudomonas corrugata* significantly improved growth parameters such as stem diameter and biomass in sorghum through the production of indole-3-acetic acid (IAA) (Amora-Lazcano et al., 2022). Another study highlighted the beneficial effects of various *Pseudomonas* strains on shoot and root biomass under salinity stress (do Nascimento et al., 2014). However, to our knowledge, no research has been conducted on the impact of *Pseudomonas stutzeri* ISE12 on enhancing sorghum plant performance under salt stress. In addition, our understanding of how strains like *P. stutzeri* ISE12 support various genotypes of the same species and their effectiveness in salt-sensitive versus salt-tolerant genotypes remains limited. This knowledge gap arises from complex host plant-microbe interactions, genetic variability within plant populations, and environmental factors such as soil salinity gradients and nutrient availability, all of which influence bioaugmentation efficacy. To fill this gap, we evaluated the effect of *P. stutzeri* ISE12 inoculation on growth and biochemical parameters of two selected sorghum genotypes that differ in salinity tolerance (salt-tolerant Pegah and salt-sensitive Payam) growth under saline conditions (Rajabi Dehnavi et al., 2020, 2023). Focusing specifically on the seed germination and seedling growth phases, we hypothesized that (1) the halotolerant plant growth-promoting endophyte *P. stutzeri* ISE12 enhances salinity tolerance in sorghum by mitigating ROS production and osmotic imbalance induced by salt stress; and (2) the degree of stimulation varies depending on the plant genotype’s sensitivity to salt stress, with salt-sensitive genotypes benefiting more significantly from the presence of this endophytic bacterium. This study represents a pioneering effort to boost sorghum using halotolerant endophytic bacteria, potentially unlocking opportunities for cultivating different sorghum genotypes under saline conditions.

## 2 Materials and methods

### 2.1 Plant material and seed preparation

According to our pervious experiments we selected two Iranian sorghum genotypes, Pegah (as salt-tolerant) and Payam (as salt-sensitive) (Rajabi Dehnavi et al., 2020, 2023). These genotypes were obtained from the Seed and Plant Improvement Institute (SPII) in Karaj, Alborz Province, Iran. For seed sterilization, seeds were immersed in a 5% sodium hypochlorite (bleach) solution in 50 mL Falcon tubes, agitated for 15 minutes, and rinsed six times with sterile distilled water. To ensure the success of the sterilization process, a sterility test of seeds and water after the last washing was conducted on petri dishes with R2A (Difco) and PDA (Difco) medium in three replications at 26°C for five days. After this time, no bacterial or fungal strain was noted.

### 2.2 Bacterial inoculum preparation

*Pseudomonas stutzeri* ISE12 (NCBI Acc. No. KX686983) was selected from a laboratory collection of halotolerant microorganisms at the Department of Microbiology, Faculty of Biological and Veterinary Sciences, Nicolaus Copernicus University in Toruń. This strain was originally isolated from the roots of the obligate halophyte Salicornia europaea and was chosen based on its plant growth-promoting properties, including the synthesis of siderophores, indolyl acetic acid, cellulases, and the presence of the *nif*H gene, indicating its ability for nitrogen fixation (Szymańska et al., 2016). Additionally, this endophytic strain has a high tolerance to salt stress in the range of 50 to 700 mM NaCl (Szymańska et al., 2020). A 3-day culture of bacteria growing on R2A medium (Difco) enriched with 2% NaCl was used to prepare the inoculum. Bacterial cells were suspended in 2% NaCl, and OD was set to 0.5 (OD is optical density, measured at 600 nm; a 0.5 value is equivalent to 1.5 x 10^8^ cells/mL). The bacterial inoculum was used twice: first, surface-sterilized seeds were incubated in the bacterial suspension for 45 minutes with shaking at 24°C, and second, 1 mL of inoculum was added into the rhizosphere of 2-week-old plants. In the case of the control variant (non-inoculated), a 2% NaCl solution was used instead of bacterial inoculum.

### 2.3 Germination experiment

The germination experiment was undertaken as a complete randomized design in 4×2×2 factorial with four replications in a growth chamber with 25°C and 16 h light period. The experiment included four salt treatments (0, 100, 150, and 200 mM NaCl), two sorghum genotypes (Pegah and Payam), and two inoculation variants (non-inoculated control and inoculated with the *P. stutzeri* ISE12 strain). Twenty-five seeds were placed in each sterile petri plate, lined with sterile Whatman No. 2 filter paper, and watered with the relevant sterile NaCl solution (5 mL for each petri dish). Then, they were monitored for ten days.

### 2.4 Germination assessment

We assessed the following parameters:

(a) germination percentage (GP)


$$GP=\;\left(\frac{n}{N}\right)\times \;100$$

where *n* is the number of normally germinated seeds and *N* is the total number of sown seeds,

(b) germination index (GI)


$$GI=\text{ Σ}\left(\frac{Gt}{Tt}\right)$$

where *Gt* is the number of seeds germinated in day t, and *Tt* is the number of days (Hakim et al., 2010),

(c) mean germination time (MGT)


$$MGT=\frac{\text{Σ}\left(Ti\times Ni\right)}{\Sigma Ni}$$

where *Ni* is the number of newly germinated seeds at time *Ti* (Alvarado et al., 1987; Ruan et al., 2002).

(d) seedling vigor (VIG)

seedling vigor (VIG) was calculated (Mahender et al., 2015):


VIG = mean germination percentage × mean seedling length

(e) seedling growth parameters

Seedlings’ growth validation was based on shoot (seSL) and root length (seRL), fresh (seFW), and dry weight (seDW). The seDW was measured after 72 h at 80°C oven drying.

### 2.5 Pot experiment

A pot experiment was similarly designed as a complete randomized design in 4×2×2 factorial with the same salt treatments. For each salinity level, 48 seeds per genotype were prepared, as described earlier. Of these, 24 seeds were planted without inoculation, and the remaining 24 seeds were planted after bacterial inoculation. All seeds were sown in sterile plastic trays, each pot with a volume of 82 cm³, filled with a sterile mixture of sand and vermiculite (volume 1:1). In total, 192 seeds were used for each genotype, encompassing all salinity levels and both inoculation conditions. Before planting, pots in each tray were saturated to their full capacity with sterile solutions of 0, 100, 150, and 200 mM NaCl. Then, tries were irrigated with sterile Hoagland medium (twice per week) and sterile distilled water (according to plant requirements) until the end of the experiment (8 weeks). Furthermore, after two weeks, bacterial suspension (1 mL per plant, 1.5 x 10^8^ cells/mL) was added to each pot to support the inoculated seeds.

### 2.6 Growth assessments

For growth assessments after eight weeks, ten plantlets were washed up from the substrate for each treatment. We measured the number of leaves (NOL), shoot and root length (SL and RL as cm), and leaf area (LA) by digishape software. The plantlet shoot (SFW) and root (RFW) fresh and dry weights (SDW and RDW, respectively) were measured, after oven drying for 72 h at 80°C. Then, we calculated some growth indexes:

(a) Specific leaf area (SLA)


$$\text{SLA }=\frac{\text{LA}}{\text{LDW}}$$

where LA is the leaf area and LDW is leaf dry weight.

(b) Leaf weight ratio (LWR)


$$\text{LWR}=\frac{\text{LDW}}{DW}$$

where LDW is the leaf dry weight and DW is the total dry weight.

(c) Relative root weight (RWR)


$$\text{RWR}=\frac{\text{RDW}}{\text{DW}}$$

where RDW is root dry weight, and DW is total dry weight.

### 2.7 Biochemical analyses

For biochemical analyses after eight weeks, three samples were used for each treatment to measure the following parameters:

#### 2.7.1 Proline content

Shoot and root proline content (SP and RP, respectively) were determined using the method described by (Bates et al., 1973). 0.5 g fresh plant samples (shoot and root separately) from each treatment were homogenized in 0.5 mL of 3% (w/v) sulphosalicylic acid. After that, 0.2 mL of each homogenate was mixed with 0.2 mL of glacial acetic acid, to which 0.2 mL of ninhydrin was added. The reaction mixture was boiled in a water bath at 100°C for 30 minutes and immediately cooled in an ice bath. After cooling, 0.4 mL of toluene was added to the reaction mixture. After thorough mixing, the chromophore containing toluene was separated, and the absorbance of the red color developed was read at 520 nm against toluene. The proline level was calculated using a standard curve (range 0-50 nmol) and expressed as nmol per gram of fresh weight (nmol/g FW).

#### 2.7.2 Peroxidase activity

Shoot, and root peroxidase enzyme activity (SPOD and RPOD) were determined following the dehydrogenation of guaiacol as a substrate, according to the method described by (Maehly, 1954). To evaluate POD activity, 0.5 g fresh plant samples (leaves, roots, and stems) were homogenized in 50 mM potassium phosphate buffer (pH 7.0), including 0.1 mM EDTA on ice in a mortar. Then, the homogenate was centrifuged at 15,000 x g for 15 minutes at 4°C. The supernatant was used to determine POD activity and protein content. The enzymatic reaction was initiated by adding 100 µl of supernatant to the mixture of 50 mM potassium phosphate buffer (pH 7.0), 20 mM guaiacol, and 40 mM H_2_O_2_. Changes in absorbance of the reaction solution at 470 nm were determined every minute. The enzyme activity was expressed as U/mg protein; U is the amount of enzyme causing a 0.001 change in absorbance per minute. The protein concentrations were assessed based on the Bradford method (Bradford, 1976). The absorbance of the protein solution was measured at 595 nm. A standard curve (range 0-1 mg/mL) was prepared from bovine serum albumin (BSA) dilutions.

#### 2.7.3 Hydrogen peroxide content

Shoot and root hydrogen peroxide content (SH_2_O_2_ and RH_2_O_2_) were measured as described by (Velikova et al., 2000). Plant tissues (500 mg) were homogenized in the ice bath with 5 mL of 0.1% trichloroacetic acid (TCA). Then the homogenate was centrifuged at 12,000 x g for 15 min (4°C), and 0.5 mL of the supernatant was added to 0.5 mL of 10 mM potassium phosphate buffer (pH 7.0) and 1 mL of 1 M potassium iodide (KI). The solution was incubated in the dark for 1 hour, and the absorbance of the supernatant was read at 390 nm. A standard curve was prepared from serial dilutions, and the final H_2_O_2_ concentration was calculated and expressed as µmol per gram of fresh weight.

### 2.8 Statistical analysis

To compare treatments, ANOVA was used with HSD, p ≤ 0.05 for *post-hoc* comparisons (Shapiro-Wilk test p > 0.05) (Statistical Analysis Software version 9.4, SAS Institute Inc., Cary, North Carolina, USA). Non-Metric Multidimensional Scaling with Bray-Curtis dissimilarity measure (NMDS, Canoco 5.0 package) was used to generalize observed effects for salt-sensitive and salt-tolerant genotypes.

## 3 Results

### 3.1 Salinity treatment and sorghum genotype interaction

**Figure 1:**
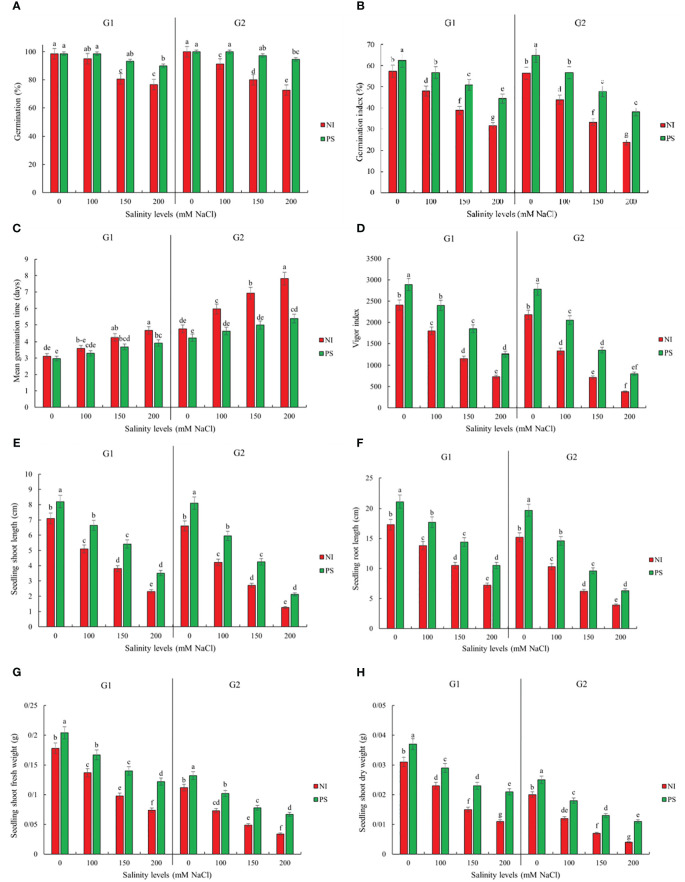
Inoculation effects on germination and seedlings traits **(A)** Germination percentage (GP), **(B)** Germination index (GI), **(C)** Mean germination time (MGT), **(D)** Vigour index (VIG), **(E)** seedling Shoot length (seSL), **(F)** seedling Root length (seRL), **(G)** Seedling fresh weight (seFW) and **(H)** Seedling dry weight (seDW) of sorghum salt-tolerant genotype Pegah (G1) and salt-sensitive Payam (G2). NI, non-inoculated control; PS, inoculated by *Pseudomonas stutzeri* ISE12. Different letters indicate significant differences by HSD at p<0.05. The comparison is done separately for each genotype. The results in the **Supplementary Table 2** are presented factorially, comparing both genotypes together.

**Figure 2:**
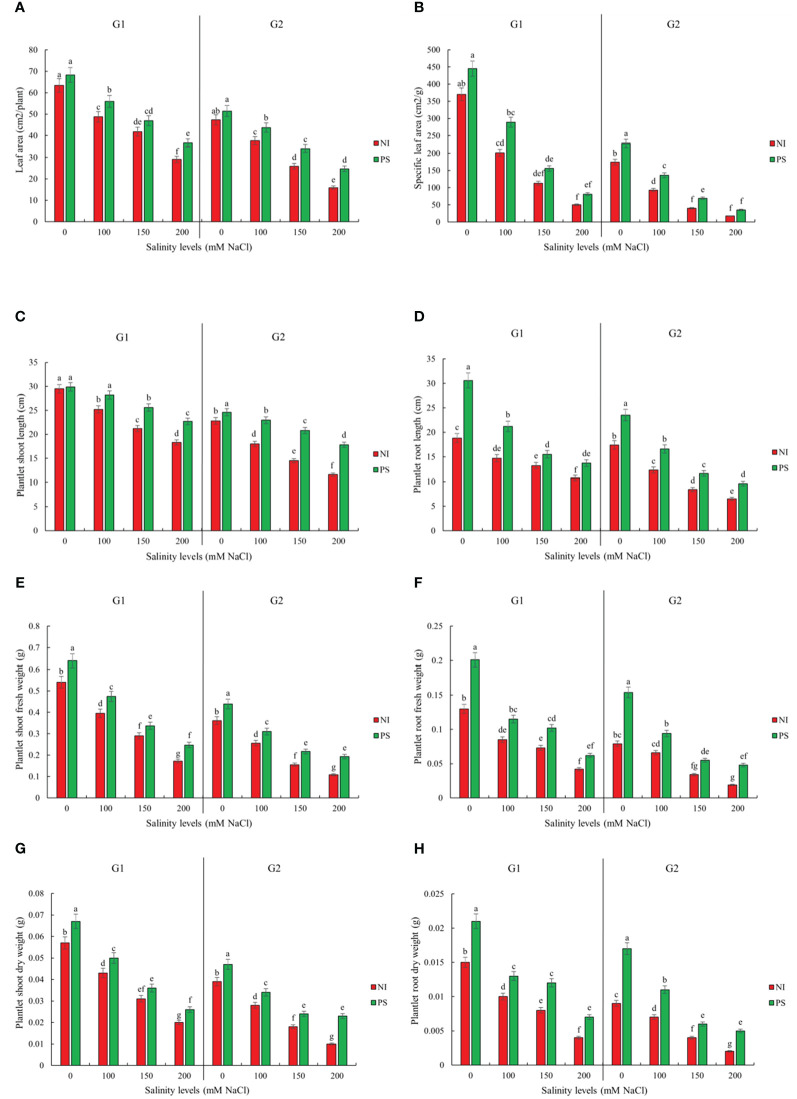
Inoculation effect on plantlets growth traits **(A)** Leaf area (LA), **(B)** Specific leaf area (SLA), **(C)** Plantlet shoot length (SL), **(D)** Plantlet root length (RL), **(E)** Plantlet shoot fresh weight (SFW), **(F)** Plantlet root fresh weight (RFW), **(G)** Plantlet shoot dry weight (SDW) and **(H)** Plantlet root dry weight (RDW) of sorghum salt-tolerant genotype Pegah (G1) and salt-sensitive Payam (G2). NI, non-inoculated control; PS, variant inoculated by *Pseudomonas stutzeri* ISE12. Different letters indicate significant differences by HSD at p<0.05. The comparison is done separately for each genotype. The results in the **Supplementary Table 3** are presented factorially, comparing both genotypes together.

**Figure 3:**
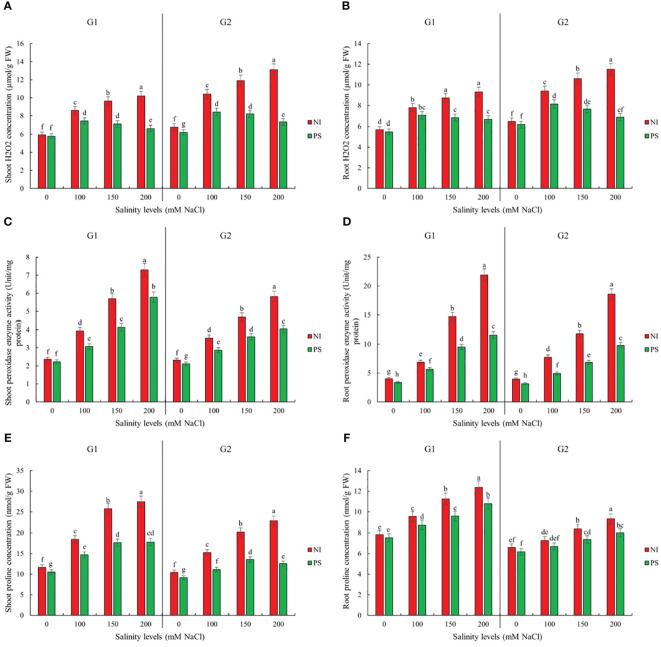
Inoculation effect on plantlets biochemical traits **(A)** Shoot H_2_O_2_ concentration (SH_2_O_2_), **(B)** Root H_2_O_2_ concentration (RH_2_O_2_), **(C)** Shoot peroxidase enzyme activity (SPOD), **(D)** Root peroxidase enzyme activity (RPOD), **(E)** Shoot proline concentration (SP) and **(F)** Root proline concentration (RP) of sorghum salt-tolerant genotype Pegah (G1) and salt-sensitive Payam (G2). NI, non-inoculated control; PS, variant inoculated by *Pseudomonas stutzeri* ISE12. Different letters indicate significant differences by HSD at p<0.05. The comparison is done separately for each genotype. The results in the **Supplementary Table 3** are presented factorially, comparing both genotypes together.

Sorghum germination, growth, and biochemical attributes significantly depended on the interaction between salinity treatment and genotype (**Supplementary Table 1**). Genotype Pegah experienced less severe reductions in various growth parameters than Payam (**Figures 1**, **2**; **Supplementary Tables 2, 3**). For example, Pegah’s germination percentage and index (GP and GI) were reduced by 1.4 times less than Payam’s, and Pegah’s mean germination time (MGT) increased by 1.4 times less than the increase observed in Payam. In addition, Pegah’s vigor index (VIG) declined 1.3 times less than the decline in Payam. Seedling shoot length (seSL) and seedling root length (seRL) in Pegah were reduced by 1.4 times lower than in Payam. Seedling fresh weight (seFW) and seedling dry weight (seDW) reductions were similarly less severe, being approximately 1.3 times lower than those in Paya (**Figure 1**; **Supplementary Table 2**). Regarding plantlets’ growth, both genotypes experienced decreases in number of leaves (NOL), shoot and root length (SL and RL), leaf area (LA), specific leaf area (SLA), shoot fresh weight (SFW), root fresh weight (RFW), shoot dry weight (SDW), and root dry weight (RDW), but the reductions were consistently more pronounced in genotype Payam (**Figure 2**; **Supplementary Table 3**). For instance, Pegah’s reductions in NOL, SL, RL, LA, SLA, SFW, RFW, SDW, and RDW were respectively about 1.5, 1.3, 1.4, 1.3, 1.1, 1.1, 1, 1.1, and 1 times less than those in Payam. Consequently, the stress indicator - hydrogen peroxide content in shoot and root (SH_2_O_2_ and RH_2_O_2_) increased respectively, 1.3 and 1.2 times less in Pegah than in Payam (**Figure 3**; **Supplementary Table 3**). Peroxidase activity in shoot and root (SPOD and RPOD) and proline in shoot and root (SP and RP) levels were also higher in Pegah, with SPOD increases up to 1.3 times greater and SP levels 1.2 times higher than Payam. However, in the case of RPOD and RP, the corresponding increases were 1.1 and 1.6 times higher in Pegah than in Payam (**Figure 3**; **Supplementary Table 3**). Overall, genotype Pegah, the salt-tolerant genotype, showed a less negative response to salinity compared to Payam, the salt-sensitive genotype, as was suggested by our previous findings (Rajabi Dehnavi et al., 2020, 2023).

### 3.2 Inoculation effects on germination and early growth stage

To evaluate the effect of salinity and inoculation with *P. stutzeri* ISE12 on germination and seedling performance in sorghum genotypes, traits related to these attributes were measured. Our results showed that salinity, inoculation, and genotype effects were significant for all evaluated traits (**Supplementary Table 1**). We observed that inoculation with *P. stutzeri* ISE12 had a beneficial impact on germination characteristics (GP, GI, MGT, and VIG) and early growth parameters (seSL, seRL, seFW, and seDW) for both sorghum genotypes under both non-saline and saline conditions (**Figure 1**; **Supplementary Table 2**). Notably, across all assessed traits and at all salinity levels, the Payam genotype showed more favorable responses to inoculation with *P. stutzeri* ISE12 than the Pegah genotype (**Figure 1**; **Supplementary Table 2**). For example, Payam’s GP increased approximately 1.8 times more than Pegah’s. Similarly, Payam’s GI improved by 1.5 times more. The reduction in MGT for Payam was two times higher than Pegah, and Payam’s VIG increased 1.5 times more than Pegah’s (**Figure 1**; **Supplementary Table 2**). Regarding seedling growth parameters, the Payam genotype also showed a better response to inoculation at all salinity levels than the Pegah genotype (**Figure 1**; **Supplementary Table 2**). For instance, Payam’s seSL improved by 23% to 68%, and seRL improved by 30% to 61%. These values represent improvements of 1.4 and 1.3 times higher, respectively, than those observed in Pegah (seSL range: 15% to 52%; seRL range: 22% to 46%). Additionally, Payam’s seFW and seDW showed increments ranging from 18% to 97% and 25% to 175%, respectively, highlighting enhancements 1.4 and 1.8 times greater than those in Pegah (seFW range: 15% to 65%; seDW range: 19% to 91%). Interestingly, the most pronounced growth enhancements for both genotypes were observed under 200 mM NaCl conditions (**Figure 1**; **Supplementary Table 2**).

### 3.3 Inoculation effects on plantlets growth

Regarding the next growth stage, our results demonstrated that the effect of inoculation was also significant for both genotypes (**Supplementary Table 1**). Notably, the application of *P. stutzeri* ISE12 markedly enhanced various growth parameters under both non-saline and saline conditions (**Figure 2**; **Supplementary Table 3**). Interestingly, the highest levels of shoot and root biomass were observed in inoculated plants under non-saline conditions (0 mM NaCl), underscoring the efficacy of *P. stutzeri* ISE12 as a biofertilizer in promoting robust plant growth (**Figure 2**; **Supplementary Table 3**). However, compared to non-inoculated controls, the most significant positive response to inoculation occurred under 200 mM NaCl. These results suggest that high salinity enhances plant-microbe interactions and causes a better plant response to inoculation. Although the final biomass accumulation was higher in the salt-tolerant genotype Pegah, the salt-sensitive genotype Payam had a higher positive response (**Figure 2**; **Supplementary Table 3**). For example, in response to inoculation along the salinity treatments, on average, Payam’s SL increased 1.5 times higher than Pegah’s. Similarly, Payam’s RL shows a 1.3 times higher increase than Pegah’s. Also, the improvement of LA in Payam was two times higher than Pegah’s. Furthermore, the SLA in Payam indicated a 1.1 times higher increase than Pegah’s. Payam’s SFW exceeded Pegah’s improvement by 1.8 times. Additionally, SDW in Payam highlights a five times higher increase than Pegah’s. RFW in Payam also demonstrated a 2.6 times higher increase than Pegah’s. Moreover, RDW in Payam was 1.3 times higher than Pegah’s range (**Figure 2**; **Supplementary Table 3**).

### 3.4 Inoculation effects on biochemical properties

Our results also show that inoculation with *P. stutzeri ISE12* causes a significant decrease in oxidative and osmotic stress expressed by SH_2_O_2_, RH_2_O_2_, SPOD, RPOD, and SP levels (**Supplementary Table 1**). We observed that the highest decrease for these biochemical parameters compared to the non-inoculated control was noted in the highest salt concentration, 200 mM NaCl (**Figure 3**; **Supplementary Table 3**). The reduction in measured biochemical parameters was higher in the case of the salt-sensitive genotype Payam (**Figure 3**; **Supplementary Table 3**). The reduction in H_2_O_2_ levels in the shoots of the Pegah genotype ranged from 3% to 35% along the salinity treatments, whereas in the Payam genotype, it was significantly greater, ranging from 9% to 44%. This result indicates that Payam experienced up to 1.26 times more H_2_O_2_ reduction in shoot than Pegah (**Figure 3**; **Supplementary Table 3**). In the roots, Pegah showed reductions between 4% and 28%, while Payam exhibited a more extensive reduction range of 5% to 40%, demonstrating that Payam had up to 1.43 times more reduction in root H_2_O_2_ levels. Regarding peroxidase enzyme activity, bacterial inoculation led to a decrease in shoot peroxidase activity by 6% to 21% in Pegah, while in Payam, the reduction was more pronounced, ranging from 9% to 30%. Payam’s reduction in shoot peroxidase activity was up to 1.43 times greater than Pegah’s (**Figure 3**; **Supplementary Table 3**). In the roots, Pegah showed a decrease in peroxidase activity ranging from 16% to 47%. In contrast, Payam experienced a slightly higher reduction range of 20% to 48%, indicating that Payam had up to 1.13 times more reduction in root peroxidase activity (**Figure 3**; **Supplementary Table 3**). The proline concentration in the shoots decreased by 10% to 35% in Pegah and by a more substantial 13% to 45% in Payam, showing that Payam had up to 1.29 times greater reduction in shoot proline concentration. In the roots, proline concentration decreased by 4% to 13% in Pegah and by a higher range of 7% to 14% in Payam, indicating that Payam experienced up to 1.75 times more reduction in root proline concentration. The results suggest that the salt-sensitive genotype has a better biochemical response to inoculation than the tolerant genotype Pegah (**Figure 3**; **Supplementary Table 3**).

### 3.5 Overall growth and development assessment

**Figure 4:**
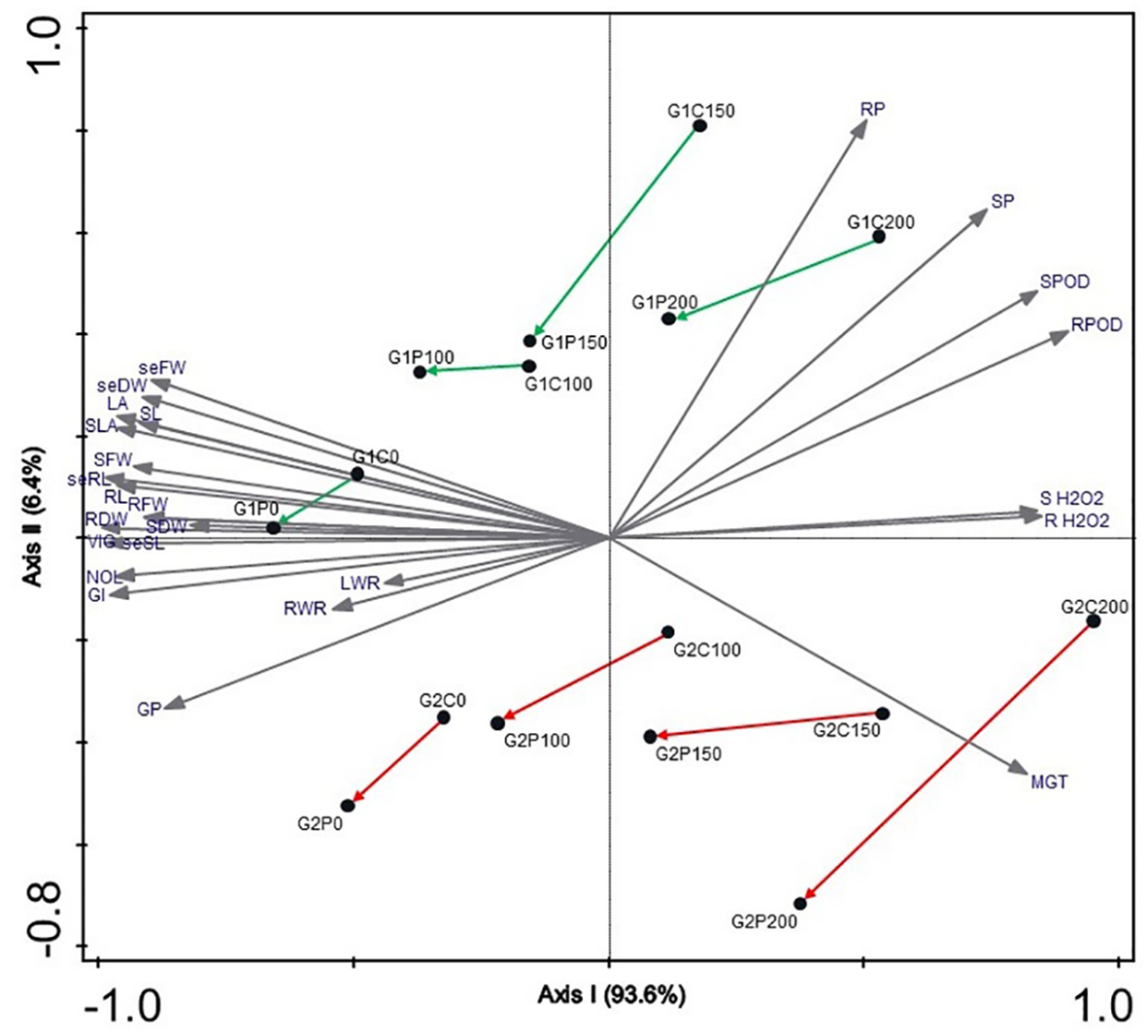
Non-metric multidimensional scaling ordination diagram (NMDS with Bray–Curtis dissimilarity measure) of two sorghum genotypes in four salinity treatments. GP, Germination percentage (%); GI, Germination index; MGT, Mean germination time; seSL, seedling Shoot length (cm); seRL, seedling Root length (cm); VIG, Vigour index; seFW, seedling Fresh weight (gr); seDW, seedling Dry weight (gr), NOL, Number of leaves; SL, Plantlet Shoot length; RL, Plantlet Root length; LA, Leaf area; SFW, Shoot Fresh weight; RFW, Root fresh weight; SDW, Root Dry weight; RDW, Root Dry weight; SLA, Specific leaf area; RWR, Root weight ratio; SH_2_O_2_, Shoot H_2_O_2_ concentration; RH_2_O_2_, Root H_2_O_2_ concentration; SPOD, Shoot peroxidase enzyme activity; RPOD, Root peroxidase enzyme activity; SP, Shoot proline concentration; RP, Root proline concentration, G1, salt-tolerant genotype Pegah, G2, salt-sensitive genotype Payam, C, non-inoculated control, P, *Pseudomonas stutzeri* ISE12 inoculation; 0, 100, 150 and 200 - salinity levels. Arrows show directions of growth improvement after inoculation in each salinity level.

The Bray-Curtis similarity between all treatments based on all included plant traits demonstrated that the salt-tolerant genotype Pegah exhibited a less negative response to salinity, but less inoculation improvement compared to the salt-sensitive genotype Payam. This distinction is clearly illustrated in the NMDS diagram, where the first ordination axis delineates the salinity gradient, accounting for 93.6% of the variance in the measured parameters (**Figure 4**). Samples are distributed along this axis from left (0 mM NaCl treatments) to right (200 mM NaCl). The second NMDS axis represents the salt tolerance of genotypes, contributing to only 6.4% of the total variance. Notably, the salt-sensitive genotype Payam is positioned in the lower part, while the salt-tolerant Pegah is positioned in the higher part of the graph. The distances between inoculated and non-inoculated controls in each salt treatment are higher for the salt-sensitive genotype, indicating better general growth performance, especially in 200 mM NaCl, than salt-tolerant.

## 4 Discussion

Our study primarily focused on investigating the responses of various sorghum genotypes to inoculation with the endophytic strain *P. stutzeri* ISE12 in saline conditions. Specifically, we aimed to assess how salt-tolerant and salt-sensitive sorghum genotypes react to this inoculation under salinity stress. Our findings reveal a consistent pattern: salinity negatively impacts various germination and growth parameters across both sorghum genotypes. This observation aligns with prior research on sorghum (Rajabi Dehnavi et al., 2020; Mansour et al., 2021; Nie et al., 2023; Rajabi Dehnavi et al., 2023), which consistently demonstrated the detrimental effects of salinity on germination and growth processes. Our research has shown that the differences between the two genotypes in response to the salinity observed at the germination stage are also reflected in the subsequent growth phase. Salinity-induced physiological drought during the germination stage reduces seed water absorption, consequently delaying germination and inhibiting seedling growth by reducing the use of seed reserves (Hasanuzzaman et al., 2013; Danial and Basset, 2024). Furthermore, physiological drought during germination and early growth stages diminishes root water absorption capacity, thus hampering plant growth and development. Additionally, it has been observed that the accumulation of ions such as Na^+^ and Cl^−^ in saline conditions disrupts various biochemical processes, including enzyme activities (Shahid et al., 2020), protein metabolism (Orcan and Orcan, 2024), phenolic compounds metabolism (Hasanuzzaman et al., 2013), hormonal balance (Cüneyt, 2020), and photosynthetic processes (Jadczak et al., 2021). This disruption and reduced transport rates of essential ions, limit the supply of crucial metabolites necessary for growth in different plant tissues, ultimately inhibiting germination and establishment (Hasanuzzaman et al., 2013; Amin et al., 2021).

Hydrogen peroxide, a potent ROS, is generated within plant cells during essential processes such as photosynthesis, photorespiration, and respiration (Smirnoff and Arnaud, 2019; Fan and Li, 2024). Our study revealed that salinity induces the production of hydrogen peroxide in both the shoot and root of sorghum genotypes, which is consistent with studies highlighting salinity-induced ROS production, including hydrogen peroxide (Balasubramaniam et al., 2023; Fan and Li, 2024). Excessive concentrations of ROS, such as H_2_O_2_, can lead to oxidative damage to membranes and apoptotic cell death (Ahmad et al., 2012; Fan and Li, 2024), thereby impeding plant growth and development by compromising membrane integrity and lipid peroxidation. Our finding shows that the salt-sensitive genotype Payam showed higher increases in SH_2_O_2_ and RH_2_O_2_, indicating a greater oxidative stress response than Pegah. This heightened oxidative stress in Payam suggests a less efficient mechanism for mitigating the detrimental effects of salinity.

Our results also indicate that increasing salt levels corresponded to heightened SPOD and RPOD activities in both genotypes, particularly in the roots. Enhanced POD activity is a protective mechanism by scavenging excess ROS generated under stress conditions, thus mitigating the adverse effects of salt stress (el Omari and Nhiri, 2015; Hasanuzzaman et al., 2020). Furthermore, biochemical analyses revealed an increase in SP and RP with rising salt levels, which proves that proline is the main osmolyte in sorghum (Temizgul et al., 2016; Ukwatta et al., 2021; Rajabi Dehnavi et al., 2023). This increase in proline content is regulated by key enzymes such as pyrroline carboxylic acid synthetase and pyrroline carboxylic acid reductase, which are involved in proline biosynthesis (Gupta and Huang, 2014; Meena et al., 2019). The higher proline content observed in the shoot than in the root suggests its potential role in stem elongation (Frimpong et al., 2021). Our results demonstrated that the salt-tolerant genotype Pegah had higher values for SPOD, RPOD, SP, and RP, reflecting a more robust antioxidative defense system and better osmotic adjustment under saline conditions. The elevated peroxidase activity and proline levels in Pegah indicate its enhanced capacity to cope with oxidative damage and maintain cellular homeostasis in the face of salt stress (Rajabi Dehnavi et al., 2023). However, it is important to note that these protective mechanisms consume energy from salt-tolerant genotypes, potentially diminishing energy efficiency for growth. Conversely, salt-sensitive genotypes may lack sufficient levels of these defense mechanisms, leading to a general reduction in plant biomass production under saline conditions.

Our study underscores the effectiveness of inoculation with *P. stutzeri* ISE12 in enhancing germination parameters across both sorghum genotypes. Consistent with our findings, previous research has demonstrated the efficacy of *P. stutzeri* ISE12 in alleviating the adverse impacts of salinity on germination in various plant species (Piernik et al., 2017; Szymańska et al., 2019, 2022). According to our results, this bacterium seems to possess mechanisms to mitigate osmotic stress induced by high salt concentrations during the initial stages of salt stress, thereby promoting greater water absorption by host plants. In addition to our results, *P. stutzeri* ISE12 is known to modulate complex signaling networks, facilitating its positive impact on plant germination under salt stress (Szymańska et al., 2016, 2019). It also seems that the ability of *P. stutzeri* ISE12 to maintain high metabolic activity in saline environments and to accumulate salt within its cells can contribute to its protective role against salinity (Piernik et al., 2017). Our study reveals that the salt-sensitive genotype showed a significantly more favorable response to bacterial inoculation than the salt-tolerant genotype in both saline and non-saline conditions. This enhanced response was most notable during germination and the initial growth of plantlets.

Our findings also demonstrate a positive impact of inoculation on growth parameters. Numerous studies have highlighted the ability of Plant Growth Promoting Bacteria (PGPBs), such as *P. stutzeri* ISE12, to enhance plant growth parameters, particularly root and shoot biomass, under salt stress conditions (Piernik et al., 2017; Szymańska et al., 2019, 2020, 2022). Specifically, it has been reported that in saline environments, *P. stutzeri* stimulates the production of gibberellins, siderophores, and other unidentified compounds, resulting in increased shoot and root length, root surface area, and root volume (Kumar Arora et al., 2020). These enhancements facilitate improved nutrient uptake by plants, promoting overall growth and development (Kumar Arora et al., 2020; Slama et al., 2023). Moreover, under saline conditions, many PGPBs exhibit the capability to mitigate the negative effects of ethylene and regulate essential phytohormone levels, thereby alleviating salt stress and facilitating plant growth (Shahzad et al., 2017; Mokrani et al., 2020; Rout et al., 2023). In addition, it is well documented that PGPBs enhance physiological processes such as carbon fixation and photosynthesis in plants experiencing salinity stress (Abd_Allah et al., 2018; Szymańska et al., 2020; Slama et al., 2023).

Our results demonstrated that *P. stutzeri* ISE12 likely mitigates oxidative stress and improves the antioxidant defense system of the plants, as indicated by reductions in SH_2_O_2_, RH_2_O_2_, SPOD, RPOD, SP, and RP levels. Several studies have demonstrated that inoculation with PGPBs can counteract the negative effects of salinity in various plant species by regulating the accumulation of osmolytes and antioxidant compounds, osmotic adjustment, cell stabilization, and free radical scavenging (Szymańska et al., 2018; Kumar Arora et al., 2020; Rout et al., 2023). Furthermore, a decrease in proline and H_2_O_2_ content in plants inoculated with PGPBs has been reported in several studies (Jha et al., 2013; Abd_Allah et al., 2018; Szymańska et al., 2019). Notably, endophytic bacteria like *P. stutzeri* not only alleviate osmotic stress in plants by enhancing water absorption but also reduce the production of ROS, particularly hydrogen peroxide, in plant tissues (Slama et al., 2023). By colonizing plant tissue, these bacteria decrease H_2_O_2_ synthesis and protect membrane lipids from peroxidation (Singh and Jha, 2016; Vaishnav et al., 2019). Moreover, *P. stutzeri* may regulate ion homeostasis in plants under saline conditions, modulating the uptake and distribution of ions, such as sodium and chloride to mitigate the negative effects of salt stress on plant physiology (Cao et al., 2023). Additionally*, P. stutzeri* may induce systemic tolerance mechanisms in plants exposed to saline stress, activating stress-responsive genes and signaling pathways to enhance the plant’s ability to withstand oxidative stress and maintain cellular homeostasis (Mishra et al., 2022). Furthermore, producing exopolysaccharides (EPS) by PGPBs such as *P. stutzeri* ISE12 can improve soil structure and water retention capacity, thereby reducing salt stress on plants (Kumar Arora et al., 2020). EPS also directly scavenges ROS and protects plant cells from oxidative damage (Kumar Arora et al., 2020). In summary, *P. stutzeri* ISE12 seems to decrease the cost required for defense mechanisms such as osmotic regulation (proline accumulation) and antioxidant defense systems (peroxidase enzyme activity), thereby fostering increased growth of sorghum plants in saline conditions.

In our thorough examination of genotype reactions to salinity and inoculation, a notable observation emerged: the salt-sensitive genotype (Payam) exhibited a significantly more positive response to *P. stutzeri* ISE12, particularly concerning the enhancement of salt tolerance. This heightened effect was discernible through a further reduction in hydrogen peroxide content, leading to a consequent decrease in proline accumulation and the activity of the antioxidant enzyme peroxidase within the salt-sensitive genotype. Significantly, this phenomenon not only facilitated the growth of salt-sensitive plants by curbing energy consumption for defense mechanisms but also effectively compensated for the inherent inefficiencies of the defense system in these plants. Our study reveals that while the salt-sensitive genotype Payam responded more positively to bacterial inoculation, the salt-tolerant genotype Pegah ultimately achieved higher dry matter accumulation, growth, and yield. Interestingly, the performance of the salt-sensitive genotype under inoculation was remarkable, with yields nearly matching those of the salt-tolerant genotype without inoculation. This finding is particularly significant, demonstrating that bacterial inoculation can enhance the resilience and productivity of salt-sensitive plants in saline conditions. This finding suggests a nuanced interplay between genotype characteristics and bacterial influence, ultimately influencing plant responses to saline conditions.

## 5 Conclusions

In conclusion, our study underscores that sorghum inoculation with halotolerant endophyte *P. stutzeri* ISE12 emerged as a significant salt stress mitigating factor. By bolstering stress tolerance mechanisms and mitigating oxidative damage, this bacterium notably enhanced germination and growth under saline conditions for both genotypes, with a particularly pronounced effect observed in the salt-sensitive Payam genotype. This approach not only preserves the inherent quality traits of salt-sensitive plants but also promises to elevate their productivity to levels akin to those of more resilient varieties. The implications of this advancement are profound, offering a potential solution for improving crop quality and expanding agricultural production in saline-affected regions while fostering sustainable farming practices. Nevertheless, the transition from laboratory findings to real-world agricultural applications warrants comprehensive field evaluations. Addressing the challenges associated with this transition will be imperative to validate the efficacy and scalability of *P. stutzeri* ISE12 as a viable strategy for saline environments.

## Data Availability

The raw data supporting the conclusions of this article will be made available by the authors, without undue reservation.
