# Supplementary material for: Mitigation of salt stress in Sorghum bicolor L. by the halotolerant endophyte Pseudomonas stutzeri ISE12
Source: Front Plant Sci. 2024 Sep 23;15:1458540. doi: 10.3389/fpls.2024.1458540 (PMC11456471; doi:10.3389/fpls.2024.1458540)
Supplement: Supplementary file 1 [file Table1.docx]

|  | **Trait** |  |  | **Sources of Variations** | | | | | |  | |
| --- | --- | --- | --- | --- | --- | --- | --- | --- | --- | --- | --- |
|  |  | **S** | **G** | | **IB** | **S×G** | **S×IB** | **G×IB** | **S×G×IB** | | **Error** |
|  | df | 3 | 1 | | 1 | 3 | 3 | 1 | 3 | |  |
|  | GP | 644.6^**^ | 3.52^n.s^ | | 1131^**^ | 4.85^n.s^ | 198.1^**^ | 63.0^*^ | 9.46^n.s^ | | 12.1 |
|  | GI | 1477^**^ | 121.5^**^ | | 1480^**^ | 32.6^**^ | 30.6^**^ | 24.9^**^ | 0.937^n.s^ | | 0.770 |
|  | MGT | 6.35^**^ | 43.4^**^ | | 12.0^**^ | 0.386^n.s^ | 0.886^*^ | 3.78^**^ | 0.241^n.s^ | | 0.182 |
| GE | seSL | 58.2^**^ | 8.87^**^ | | 23.0^**^ | 0.522^*^ | 0.231^*^ | 0.006^n.s^ | 0.076^n.s^ | | 0.077 |
|  | seRL | 288.1^**^ | 132.4^**^ | | 162.2^**^ | 4.11^**^ | 1.078^*^ | 0.028^n.s^ | 0.424^n.s^ | | 0.240 |
|  | VIG | 7103598^**^ | 1586579^**^ | | 4106460^**^ | 53818^*^ | 26366^*^ | 1122^n.s^ | 10621^n.s^ | | 8249 |
|  | seFW | 0.015^**^ | 0.042^**^ | | 0.012^**^ | 0.0003^*^ | 0.0002^*^ | 0.00003^n.s^ | 0.00003^n.s^ | | 0.00004 |
|  | seDW | 0.0003^**^ | 0.001^**^ | | 0.0005^**^ | 0.000006^*^ | 0.000005^*^ | 0.000006^*^ | 0.000001^n.s^ | | 0.000001 |
|  | NOL | 9.77^**^ | 2.25^*^ | | 6.81^**^ | 0.089^n.s^ | 0.273^n.s^ | 0.006^n.s^ | 0.139^n.s^ | | 0.231 |
|  | SL | 621^**^ | 1407^**^ | | 583^**^ | 0.430^n.s^ | 48.2^**^ | 38.6^*^ | 0.051^n.s^ | | 4.69 |
|  | RL | 1201^**^ | 662^**^ | | 1021^**^ | 1.31^n.s^ | 82.0^**^ | 30.2^*^ | 20.9^*^ | | 4.63 |
|  | LA | 6991^**^ | 7587^**^ | | 1675^**^ | 46.9^n.s^ | 22.3^n.s^ | 4.14^n.s^ | 9.27^n.s^ | | 38.3 |
|  | SFW | 0.756^**^ | 0.697^**^ | | 0.208^**^ | 0.029^**^ | 0.002^n.s^ | 0.0002^n.s^ | 0.001^n.s^ | | 0.0019 |
|  | RFW | 0.071^**^ | 0.042^**^ | | 0.057^**^ | 0.002^*^ | 0.005^**^ | 0.000002^n.s^ | 0.0002^n.s^ | | 0.0005 |
|  | SDW | 0.008^**^ | 0.007^**^ | | 0.002^**^ | 0.0002^**^ | 0.00003^n.s^ | 0.0000008^n.s^ | 0.00004^n.s^ | | 0.00002 |
|  | RDW | 0.0008^**^ | 0.0005^**^ | | 0.0006^**^ | 0.00003^**^ | 0.00003^**^ | 0.00000002^n.s^ | 0.000005^n.s^ | | 0.000002 |
| PE | SLA | 510638^**^ | 516948^**^ | | 89165^**^ | 52915^**^ | 4576^n.s^ | 5866^n.s^ | 514^n.s^ | | 6490 |
|  | LWR | 0.0003^n.s^ | 0.0005^n.s^ | | 0.001^n.s^ | 0.001^n.s^ | 0.0002^n.s^ | 0.0002^n.s^ | 0.001^n.s^ | | 0.0007 |
|  | RWR | 0.013^*^ | 0.0000004^n.s^ | | 0.090^**^ | 0.017^*^ | 0.010^*^ | 0.00006^n.s^ | 0.007^n.s^ | | 0.002 |
|  | SH_2_O_2_ | 26.02^**^ | 22.85^**^ | | 71.59^**^ | 0.830^**^ | 10.53^**^ | 3.93^**^ | 0.435^*^ | | 0.0654 |
|  | RH_2_O_2_ | 18.1^**^ | 15.9^**^ | | 40.1^**^ | 2.276^*^ | 6.79^**^ | 2.45^*^ | 0.494^n.s^ | | 0.2469 |
|  | SPOD | 27.07^**^ | 5.65^**^ | | 11.3^**^ | 1.36^**^ | 1.24^**^ | 0.023^n.s^ | 0.076^n.s^ | | 0.0126 |
|  | RPOD | 323^**^ | 21.9^**^ | | 225^**^ | 6.85^**^ | 45.9^**^ | 0.00007^n.s^ | 1.241^**^ | | 0.0444 |
|  | SP | 240^**^ | 157^**^ | | 379^**^ | 8.58^**^ | 44.0^**^ | 0.012^n.s^ | 0.659^n.s^ | | 0.1791 |
|  | RP | 21.4^**^ | 59.8^**^ | | 11.1^**^ | 1.43^*^ | 0.808^n.s^ | 0.176^n.s^ | 0.073^n.s^ | | 0.165 |
|  |  |  |  |  |  |  |  |  |  |  |  |

**Supplementary Table 1.** Analysis of variances (mean squares) for different parameters of two sorghum genotypes (Pegah and Payam) in four levels salinity and inoculation with *P. stutzeri* in germination experiment (GE) and pot experiment (PE).

GP= germination percentage; GI= germination index; MGT= mean germination time; seSL= seedling shoot length; seRL= seedling root length; VIG= vigor index; seFW= seedling fresh weight; seDW= seedling dry weight; NOL= Number of leaves; SL= plantlet hoot length; RL= plantlet root length; LA= leaf area; SFW= shoot fresh weight; RFW= root fresh weight; SDW= soot dry weight; RDW= Root Dry weight; SLA= Specific leaf area; LWR= Leaf weight ratio; RWR= Root weight ratio; SH_2_O_2_= shoot H_2_O_2_ concentration; RH_2_O_2_= root H_2_O_2_ concentration; SPOD= shoot peroxidase enzyme activity; RPOD= root peroxidase enzyme activity; SP= shoot proline concentration; RP= root proline concentration; S = salinity; G = sorghum genotype; IB= inoculation with bacteria; df = degrees of freedom; n.s = non-significant; Error = within group variance; * = *P* ≤ 0.05; ** = *P* ≤ 0.01.
