# Supplementary material for: Mitigation of salt stress in Sorghum bicolor L. by the halotolerant endophyte Pseudomonas stutzeri ISE12
Source: Front Plant Sci. 2024 Sep 23;15:1458540. doi: 10.3389/fpls.2024.1458540 (PMC11456471; doi:10.3389/fpls.2024.1458540)
Supplement: Supplementary file 2 [file Table2.docx]

**Supplementary Table 2.** Effect of salinity (0, 100, 150 and 200 mM NaCl concentration), sorghum genotypes (Pegah and Payam) and Inoculation with *P. stutzeri* ISE12 on different parameters in germination experiment (GE).

|  |  |  |  |  |  |  | **SALT LEVELS mM NaCl** | | | |  |  |
| --- | --- | --- | --- | --- | --- | --- | --- | --- | --- | --- | --- | --- |
|  | **Trait** |  | **GENOTYPE** | **INOCULATION** |  | **0** | | **100** | **150** | **200** | | |
|  |  |  |  | NI |  | 98.6 ^ab^ | | 95 ^abc^ | 80.6 ^d^ | 76.6 ^de^ | | |
|  |  |  | Pegah | PS |  | 98.6 ^ab^ | | 98.6 ^ab^ | 93.3 ^bc^ | 90 ^c^ | | |
|  | GP |  |  | Changes (%) |  | (0) | | (+4) | (+16) | (+18) | | |
|  |  |  |  | NI |  | 100 ^a^ | | 91.3 ^c^ | 80 ^d^ | 72.6 ^e^ | | |
|  |  |  | Payam | PS |  | 100 ^a^ | | 100 ^a^ | 97.3 ^ab^ | 94.6 ^ab^ | | |
|  |  |  |  | Changes (%) |  | (0) | | (+10) | (+22) | (+30) | | |
|  |  |  |  | NI |  | 57.3 ^c^ | | 48.1 ^e^ | 38.9 ^g^ | 31.5 ^i^ | | |
|  |  |  | Pegah | PS |  | 62.4 ^b^ | | 56.7 ^c^ | 50.9 ^d^ | 44.4 ^f^ | | |
|  | GI |  |  | Changes (%) |  | (+9) | | (+18) | (+31) | (+41) | | |
|  |  |  |  | NI |  | 56.4 ^c^ | | 43.9 ^f^ | 33.3 ^h^ | 23.7 ^j^ | | |
|  |  |  | Payam | PS |  | 64.8 ^a^ | | 56.7 ^c^ | 47.8 ^e^ | 38.2 ^g^ | | |
|  |  |  |  | Changes (%) |  | (+15) | | (+29) | (+43) | (+61) | | |
|  |  |  |  | NI |  | 3.12 ^jk^ | | 3.59 ^hijk^ | 4.25 ^fgh^ | 4.66 ^ef^ | | |
|  |  |  | Pegah | PS |  | 2.97 ^k^ | | 3.29 ^ijk^ | 3.68 ^hij^ | 3.92 ^ghi^ | | |
|  | MGT |  |  | Changes (%) |  | (-5) | | (-8) | (-13) | (-16) | | |
|  |  |  |  | NI |  | 4.75 ^def^ | | 5.97 ^c^ | 6.93 ^b^ | 7.82 ^a^ | | |
|  |  |  | Payam | PS |  | 4.23 ^fgh^ | | 4.62 ^efg^ | 4.99 ^de^ | 5.38 ^cd^ | | |
|  |  |  |  | Changes (%) |  | (-11) | | (-23) | (-28) | (-31) | | |
|  |  |  |  | NI |  | 7.10 ^b^ | | 5.10 ^e^ | 3.81 ^fg^ | 2.31 ^hi^ | | |
|  |  |  | Pegah | PS |  | 8.20 ^a^ | | 6.64 ^bc^ | 5.42 ^e^ | 3.51 ^g^ | | |
|  | seSL |  |  | Changes (%) |  | (+15) | | (+30) | (+42) | (+52) | | |
|  |  |  |  | NI |  | 6.60 ^c^ | | 4.22 ^f^ | 2.71 ^h^ | 1.26 ^j^ | | |
|  |  |  | Payam | PS |  | 8.10 ^a^ | | 5.95 ^d^ | 4.25 ^f^ | 2.12 ^i^ | | |
|  |  |  |  | Changes (%) |  | (+23) | | (+41) | (+57) | (+68) | | |
|  |  |  |  | NI |  | 17.3 ^c^ | | 13.8 ^e^ | 14.4 ^de^ | 7.2 ^h^ | | |
|  |  |  | Pegah | PS |  | 21.1 ^a^ | | 17.7 ^c^ | 10.5 ^f^ | 10.5 ^f^ | | |
|  | seRL |  |  | Changes (%) |  | (+22) | | (+28) | (+27) | (+46) | | |
|  |  |  |  | NI |  | 15.2 ^d^ | | 10.3 ^fg^ | 6.2 ^i^ | 3.9 ^j^ | | |
|  |  |  | Payam | PS |  | 19.7 ^b^ | | 14.6 ^de^ | 9.6 ^g^ | 6.3 ^i^ | | |
|  |  |  |  | Changes (%) |  | (+30) | | (+42) | (+55) | (+61) | | |
|  |  |  |  | NI |  | 2407 ^b^ | | 1798 ^d^ | 1158 ^f^ | 731 ^g^ | | |
|  |  |  | Pegah | PS |  | 2889 ^a^ | | 2399 ^b^ | 1850 ^d^ | 1257 ^ef^ | | |
|  | VIG |  |  | Changes (%) |  | (+20) | | (+33) | (+60) | (+72) | | |
|  |  |  |  | NI |  | 2180 ^c^ | | 1327 ^e^ | 714 ^g^ | 380 ^h^ | | |
|  |  |  | Payam | PS |  | 2780 ^a^ | | 2053 ^c^ | 1346 ^e^ | 801 ^g^ | | |
|  |  |  |  | Changes (%) |  | (+28) | | (+55) | (+89) | (+111) | | |
|  |  |  |  | NI |  | 0.178 ^b^ | | 0.137 ^d^ | 0.098 ^h^ | 0.074 ^ij^ | | |
|  |  |  | Pegah | PS |  | 0.204 ^a^ | | 0.167 ^c^ | 0.140 ^d^ | 0.122 ef | | |
|  | seFW |  |  | Changes (%) |  | (+15) | | (+22) | (+43) | (+65) | | |
|  |  |  |  | NI |  | 0.112 ^fg^ | | 0.073 ^ij^ | 0.049 ^k^ | 0.034 ^l^ | | |
|  |  |  | Payam | PS |  | 0.132 ^de^ | | 0.102 ^gh^ | 0.078 ^i^ | 0.067 ^j^ | | |
|  |  |  |  | Changes (%) |  | (+18) | | (+40) | (+59) | (+97) | | |
|  |  |  |  | NI |  | 0.031 ^b^ | | 0.023 ^e^ | 0.015 ^h^ | 0.011 ^j^ | | |
|  |  |  | Pegah | PS |  | 0.037 ^a^ | | 0.029 ^c^ | 0.023 ^e^ | 0.021 ^f^ | | |
|  | seDW |  |  | Changes (%) |  | (+19) | | (+26) | (+53) | (+91) | | |
|  |  |  |  | NI |  | 0.020 ^f^ | | 0.012 ^ij^ | 0.007 ^k^ | 0.004 ^l^ | | |
|  |  |  | Payam | PS |  | 0.025 ^d^ | | 0.018 ^g^ | 0.013 ^i^ | 0.011 ^j^ | | |
|  |  |  |  | Changes (%) |  | (+25) | | (+50) | (+85) | (+175) | | |

NI – non inoculated control, PS – variant inoculated by *Pseudomonas stutzeri* ISE12. GP= germination percentage (%); GI= germination index; MGT= mean germination time (days); seSL= seedling shoot length (cm); seRL= seedling root length (cm); VIG= vigor index; seFW= seedling fresh weight (g), seDW= seedling dry weight (g). Values within a group in each trait bearing different superscripts are significantly different at p ≤ 0.05. The numbers in Parentheses represent the percentage of changes compare to NI control.
