# Supplementary material for: Mitigation of salt stress in Sorghum bicolor L. by the halotolerant endophyte Pseudomonas stutzeri ISE12
Source: Front Plant Sci. 2024 Sep 23;15:1458540. doi: 10.3389/fpls.2024.1458540 (PMC11456471; doi:10.3389/fpls.2024.1458540)
Supplement: Supplementary file 3 [file Table3.docx]

**Supplementary Table 3.** Effect of salinity (0, 100, 150 and 200 mM NaCl concentration), sorghum genotypes (Pegah and Payam) and inoculation with *Pseudomonas stutzeri* ISE12 on different parameters in pot experiment.

|  |  |  |  |  |  |  | **SALT LEVELS mM NaCl** | | | |  |  |
| --- | --- | --- | --- | --- | --- | --- | --- | --- | --- | --- | --- | --- |
|  | **Trait** |  | **GENOTYPE** | **INOCULATION** |  | **0** | | **100** | **150** | **200** | | |
|  |  |  |  | NI |  | 5.1 ^b^ | | 4.9 ^bc^ | 4.3 ^d^ | 4.2 ^de^ | | |
|  |  |  | Pegah | PS |  | 5.7 ^a^ | | 5.0 ^b^ | 4.9 ^bc^ | 4.5 ^cd^ | | |
|  | NOL |  |  | Changes (%) |  | (+12) | | (+2) | (+14) | (+7) | | |
|  |  |  |  | NI |  | 5.0 ^b^ | | 4.5 ^cd^ | 4.2 ^de^ | 3.8 ^e^ | | |
|  |  |  | Payam | PS |  | 5.6 ^a^ | | 4.8 ^bc^ | 4.5 ^cd^ | 4.3 ^d^ | | |
|  |  |  |  | Changes (%) |  | (+12) | | (+7) | (+7) | (+13) | | |
|  |  |  |  | NI |  | 29.5 ^a^ | | 25.2 ^b^ | 21.2 ^de^ | 18.3 ^f^ | | |
|  |  |  | Pegah | PS |  | 29.9 ^a^ | | 28.2 ^a^ | 25.6 ^b^ | 22.7 ^de^ | | |
|  | SL |  |  | Changes (%) |  | (+2) | | (+12) | (+21) | (+24) | | |
|  |  |  |  | NI |  | 22.8 ^cd^ | | 18.0 ^f^ | 14.5 ^g^ | 11.6 ^h^ | | |
|  |  |  | Payam | PS |  | 24.6 ^bc^ | | 22.9 ^cd^ | 20.8 ^e^ | 17.8 ^f^ | | |
|  |  |  |  | Changes (%) |  | (+8) | | (+27) | (+43) | (+53) | | |
|  |  |  |  | NI |  | 18.8 ^d^ | | 14.7 ^fgh^ | 13.2 ^hij^ | 10.7 ^kl^ | | |
|  |  |  | Pegah | PS |  | 30.6 ^a^ | | 21.2 ^c^ | 15.5 ^efg^ | 13.7 ^ghi^ | | |
|  | RL |  |  | Changes (%) |  | (+63) | | (+44) | (+18) | (+28) | | |
|  |  |  |  | NI |  | 17.4 ^de^ | | 12.3 ^ijk^ | 8.4 ^m^ | 6.5 ^n^ | | |
|  |  |  | Payam | PS |  | 23.5 ^b^ | | 16.6 ^ef^ | 11.6 ^jk^ | 9.6 ^lm^ | | |
|  |  |  |  | Changes (%) |  | (+35) | | (+35) | (+38) | (+48) | | |
|  |  |  |  | NI |  | 63.4 ^a^ | | 48.7 ^cd^ | 41.8 ^ef^ | 29.1 ^hi^ | | |
|  |  |  | Pegah | PS |  | 68.3 ^a^ | | 55.9 ^b^ | 46.9 ^cde^ | 36.5 ^fg^ | | |
|  | LA |  |  | Changes (%) |  | (+8) | | (+15) | (+12) | (+25) | | |
|  |  |  |  | NI |  | 47.2 ^cde^ | | 37.6 ^fg^ | 25.9 ^i^ | 15.8 ^j^ | | |
|  |  |  | Payam | PS |  | 51.4 ^bc^ | | 43.7 ^de^ | 34.1 ^gh^ | 24.7 ^i^ | | |
|  |  |  |  | Changes (%) |  | (+9) | | (+16) | (+32) | (+56) | | |
|  |  |  | Pegah | NI |  | 0.540 ^b^ | | 0.395 ^d^ | 0.290 ^gh^ | 0.172 ^l^ | | |
|  |  |  |  | PS |  | 0.639 ^a^ | | 0.474 ^c^ | 0.337 ^ef^ | 0.247 ^ij^ | | |
|  | SFW |  |  | Changes (%) |  | (+18) | | (+20) | (+16) | (+44) | | |
|  |  |  | Payam | NI |  | 0.361 ^de^ | | 0.256 ^hi^ | 0.155 ^l^ | 0.108 ^m^ | | |
|  |  |  |  | PS |  | 0.439 ^c^ | | 0.310 ^fg^ | 0.217 ^jk^ | 0.193 ^kl^ | | |
|  |  |  |  | Changes (%) |  | (+22) | | (+21) | (+40) | (+79) | | |
|  |  |  | Pegah | NI |  | 0.057 ^b^ | | 0.043 ^de^ | 0.031 ^hi^ | 0.020 ^lm^ | | |
|  |  |  |  | PS |  | 0.067 ^a^ | | 0.050 ^c^ | 0.036 ^fg^ | 0.026 ^jk^ | | |
|  | SDW |  |  | Changes (%) |  | (+18) | | (+16) | (+16) | (+30) | | |
|  |  |  | Payam | NI |  | 0.039 ^ef^ | | 0.028 ^ij^ | 0.018 ^m^ | 0.010 ^n^ | | |
|  |  |  |  | PS |  | 0.047 ^cd^ | | 0.034 ^gh^ | 0.024 ^jkl^ | 0.023 ^kl^ | | |
|  |  |  |  | Changes (%) |  | (+21) | | (+21) | (+33) | (+130) | | |
|  |  |  |  | NI |  | 0.130 ^c^ | | 0.085 ^efg^ | 0.073 ^ghi^ | 0.042 ^kl^ | | |
|  |  |  | Pegah | PS |  | 0.201 ^a^ | | 0.115 ^cd^ | 0.102 ^de^ | 0.062 ^h-k^ | | |
|  | RFW |  |  | Changes (%) |  | (+55) | | (+35) | (+40) | (+48) | | |
|  |  |  |  | NI |  | 0.079 ^fgh^ | | 0.066 ^g-j^ | 0.034 ^lm^ | 0.019 ^m^ | | |
|  |  |  | Payam | PS |  | 0.154 ^b^ | | 0.094 ^ef^ | 0.055 ^i-l^ | 0.048 ^jkl^ | | |
|  |  |  |  | Changes (%) |  | (+95) | | (+42) | (+62) | (+153) | | |
|  |  |  |  | NI |  | 0.015 ^c^ | | 0.010 ^f^ | 0.008 ^gh^ | 0.004 ^lm^ | | |
|  |  |  | Pegah | PS |  | 0.021 ^a^ | | 0.013 ^d^ | 0.012 ^de^ | 0.007 ^ij^ | | |
|  | RDW |  |  | Changes (%) |  | (+40) | | (+30) | (+50) | (+75) | | |
|  |  |  |  | NI |  | 0.009 ^fg^ | | 0.007 ^hi^ | 0.004 ^m^ | 0.002 ^n^ | | |
|  |  |  | Payam | PS |  | 0.017 ^b^ | | 0.011 ^e^ | 0.006 ^jk^ | 0.005 ^kl^ | | |
|  |  |  |  | Changes (%) |  | (+89) | | (+57) | (+50) | (+150) | | |
|  |  |  |  | NI |  | 370 ^b^ | | 201 ^de^ | 113 ^g-f^ | 50 ^ijk^ | | |
|  |  |  | Pegah | PS |  | 445 ^a^ | | 289 ^c^ | 156 ^efg^ | 81 ^h-k^ | | |
|  | SLA |  |  | Changes (%) |  | (+20) | | (+44) | (+38) | (+62) | | |
|  |  |  |  | NI |  | 174 ^def^ | | 93 ^g-j^ | 40 ^jk^ | 17 ^k^ | | |
|  |  |  | Payam | PS |  | 228 ^cd^ | | 136 ^g-f^ | 69 ^h-k^ | 35 ^jk^ | | |
|  |  |  |  | Changes (%) |  | (+31) | | (+46) | (+72) | (+105) | | |
|  |  |  |  |  |  |  | |  |  |  | | |
|  |  |  |  |  |  |  | |  |  |  | | |
|  |  |  |  |  |  |  | |  |  |  | | |
|  |  |  |  | NI |  | 0.243 ^a^ | | 0.236 ^ab^ | 0.228 ^ab^ | 0.240 ^a^ | | |
|  |  |  | Pegah | PS |  | 0.244 ^a^ | | 0.249 ^a^ | 0.227 ^ab^ | 0.237 ^ab^ | | |
|  | LWR |  |  | Changes (%) |  | (+0.4) | | (+5) | (-0.4) | (-1) | | |
|  |  |  |  | NI |  | 0.231 ^ab^ | | 0.234 ^ab^ | 0.243 ^a^ | 0.215 ^b^ | | |
|  |  |  | Payam | PS |  | 0.243 ^a^ | | 0.226 ^ab^ | 0.244 ^a^ | 0.242 ^a^ | | |
|  |  |  |  | Changes (%) |  | (+5) | | (-3) | (+0.4) | (+12) | | |
|  |  |  |  | NI |  | 0.269 ^b^ | | 0.233 ^b^ | 0.267 ^b^ | 0.237 ^b^ | | |
|  |  |  | Pegah | PS |  | 0.328 ^a^ | | 0.264 ^b^ | 0.333 ^a^ | 0.264 ^b^ | | |
|  | RWR |  |  | Changes (%) |  | (+22) | | (+13) | (+25) | (+11) | | |
|  |  |  |  | NI |  | 0.237 ^b^ | | 0.263 ^b^ | 0.245 ^b^ | 0.254 ^b^ | | |
|  |  |  | Payam | PS |  | 0.353 ^a^ | | 0.338 ^a^ | 0.254 ^b^ | 0.250 ^b^ | | |
|  |  |  |  | Changes (%) |  | (+49) | | (+28) | (+4) | (-1) | | |
|  |  |  |  | NI |  | 5.95 ^ij^ | | 8.59 ^e^ | 9.62 ^d^ | 10.2 ^c^ | | |
|  |  |  | Pegah | PS |  | 5.78 ^j^ | | 7.42 ^e^ | 7.09 ^fg^ | 6.64 ^h^ | | |
|  | SH_2_O_2_ |  |  | Changes (%) |  | (-3) | | (-14) | (-26) | (-35) | | |
|  |  |  |  | NI |  | 6.80 ^gh^ | | 10.4 ^c^ | 11.9 ^b^ | 13.1 ^a^ | | |
|  |  |  | Payam | PS |  | 6.21 ^i^ | | 8.41 ^e^ | 8.21 ^e^ | 7.32 ^f^ | | |
|  |  |  |  | Changes (%) |  | (-9) | | (-19) | (-31) | (-44) | | |
|  |  |  |  | NI |  | 5.70 ^jk^ | | 7.78 ^ef^ | 8.73 ^cd^ | 9.31 ^c^ | | |
|  |  |  | Pegah | PS |  | 5.49 ^k^ | | 7.04 ^fgh^ | 6.82 ^hi^ | 6.66 ^hi^ | | |
|  | RH_2_O_2_ |  |  | Changes (%) |  | (-4) | | (-10) | (-22) | (-28) | | |
|  |  |  |  | NI |  | 6.46 ^hij^ | | 9.39 ^c^ | 10.6 ^b^ | 11.5 ^a^ | | |
|  |  |  | Payam | PS |  | 6.15 ^ijk^ | | 8.14 ^de^ | 7.65 ^efg^ | 6.87 ^ghi^ | | |
|  |  |  |  | Changes (%) |  | (-5) | | (-13) | (-28) | (-40) | | |
|  |  |  |  | NI |  | 2.36 ^i^ | | 3.93 ^e^ | 5.69 ^b^ | 7.29 ^a^ | | |
|  |  |  | Pegah | PS |  | 2.22 ^ij^ | | 3.07 ^g^ | 4.14 ^d^ | 5.78 ^b^ | | |
|  | SPOD |  |  | Changes (%) |  | (-6) | | (-22) | (-27) | (-21) | | |
|  |  |  |  | NI |  | 2.31 ^i^ | | 3.54 ^f^ | 4.68 ^c^ | 5.82 ^b^ | | |
|  |  |  | Payam | PS |  | 2.11 ^j^ | | 2.87 ^h^ | 3.61 ^f^ | 4.05 ^de^ | | |
|  |  |  |  | Changes (%) |  | (-9) | | (-19) | (-23) | (-30) | | |
|  |  |  |  | NI |  | 4.02 ^j^ | | 6.89 ^g^ | 14.7 ^c^ | 21.89 ^a^ | | |
|  |  |  | Pegah | PS |  | 3.38 ^k^ | | 5.66 ^h^ | 9.52 ^e^ | 11.58 ^d^ | | |
|  | RPOD |  |  | Changes (%) |  | (-16) | | (-18) | (-35) | (-47) | | |
|  |  |  |  | NI |  | 3.94 ^j^ | | 7.75 ^f^ | 11.81 ^d^ | 18.57 ^b^ | | |
|  |  |  | Payam | PS |  | 3.15 ^k^ | | 4.92 ^i^ | 6.86 ^g^ | 9.80 ^e^ | | |
|  |  |  |  | Changes (%) |  | (-20) | | (-37) | (-42) | (-48) | | |
|  |  |  |  | NI |  | 11.65 ^j^ | | 18.46 ^e^ | 25.77 ^b^ | 27.42 ^a^ | | |
|  |  |  | Pegah | PS |  | 10.54 ^k^ | | 14.67 ^g^ | 17.66 ^f^ | 17.80 ^ef^ | | |
|  | SP |  |  | Changes (%) |  | (-10) | | (-21) | (-31) | (-35) | | |
|  |  |  |  | NI |  | 10.44 ^k^ | | 15.23 ^g^ | 20.22 ^d^ | 22.81 ^c^ | | |
|  |  |  | Payam | PS |  | 9.13 ^l^ | | 11.10 ^jk^ | 13.55 ^h^ | 12.54 ^i^ | | |
|  |  |  |  | Changes (%) |  | (-13) | | (-27) | (-33) | (-45) | | |
|  |  |  |  | NI |  | 7.84 ^fgh^ | | 9.55 ^c^ | 11.26 ^b^ | 12.37 ^a^ | | |
|  |  |  | Pegah | PS |  | 7.54 ^gh^ | | 8.76 ^de^ | 9.59 ^c^ | 10.80 ^b^ | | |
|  | RP |  |  | Changes (%) |  | (-4) | | (-8) | (-15) | (-13) | | |
|  |  |  |  | NI |  | 6.60 ^j^ | | 7.28 ^hi^ | 8.40 ^ef^ | 9.32 ^cd^ | | |
|  |  |  | Payam | PS |  | 6.17 ^j^ | | 6.69 ^ij^ | 7.36 ^ghi^ | 8.02 ^fg^ | | |
|  |  |  |  | Changes (%) |  | (-7) | | (-8) | (-12) | (-14) | | |

NI – non inoculated control, PS – variant inoculated by *Pseudomonas stutzeri* ISE12. NOL= Number of leaves; SL= shoot length (cm); RL= root length (cm); LA= leaf area (cm^2^); SFW= shoot Fresh weight (g); RFW= root fresh weight (g); SDW= soot dry weight (g); RDW= root dry weight (g); SLA= specific leaf area (cm^2^/g) ; LWR= leaf weight ratio; RWR= root weight ratio; SH_2_O_2_= shoot H_2_O_2_ concentration (µmol/g FW); RH_2_O_2_= root H_2_O_2_ concentration (µmol/g FW); SPOD= shoot peroxidase enzyme activity (U/mg protein); RPOD= root peroxidase enzyme activity (U/mg protein); SP= shoot proline concentration (nmol/g FW); RP= root proline concentration (nmol/g FW). Values within a group in each trait bearing different superscripts are significantly different at p ≤ 0.05. The numbers in Parentheses represent the percentage of changes compare to NI control.
